# Constituent power and its institutions

**DOI:** 10.1057/s41296-021-00467-z

**Published:** 2021-04-06

**Authors:** Joel I. Colón-Ríos, Eva Marlene Hausteiner, Hjalte Lokdam, Pasquale Pasquino, Lucia Rubinelli, William Selinger

**Affiliations:** 1 Victoria University of Wellington, GB 302, Wellington, New Zealand; 2 University of Bonn, 2553113 Bonn, Germany; 3 London School of Economics and Political Science, London, WC2A 2AE UK; 4 New York University, New York, NY 10012 USA; 5 University of Cambridge, Cambridge, CB3 9AN UK; 6 University College London, London, WC1E 6BT UK

At least since the French Revolution, the idea of constituent power has been used to indicate the power the people have to create legal-political orders. As such, the history of constituent power is deeply tied to the principle of popular power and, through it, to the history of democracy, to its theory and to its practice. Not only does constituent power point to the process through which a democratic polity is instituted via procedures of constitution-making. It also acts as a reminder that the source of constitutional normativity lies in the will of the people. As a result, constituent power functions as a ‘bridge concept’ between the sphere of law and that of politics. This has traditionally resulted in two separate fields of academic scholarship. On the one side are those who think about constituent power to study the legal implications of the idea. They tend to focus on the workings of constituent assemblies and on the status of constitutional norms, their amendment procedures and their relationship to secondary law-making. This approach highlights the centrality of the idea of constituent power to the workings of the legal system. On the other side, are those scholars who look at constituent power to emphasise its political dimension. This is often part of a project of constitutional contestation, whereby constituent power is presented as an absolute popular power that can never be reduced to legal norms and that, as a result, exists alongside the constitutional system and is always potentially capable of overturning it. In this critical exchange, we aim to raise a different set of questions and ask how the idea of constituent power informs our way of thinking about some fundamental institutions of the modern democratic state: constitutional courts, legislatures, federalism, central banks and referendums.

At its core, the legitimacy of modern democratic states lies in the fact that they are structured around the principle according to which power belongs to, and springs from, the will of the people. This is evident at the level of the state’s structure, and much work has been done to determine the specific relationship that ties the liberal constitutional state to the democratic principle of popular power. Moreover, the legitimacy of single institutions depends on their consistency with the principle of popular power. This is not only true of well-studied institutions such as parliaments and constitutional courts, but also of relatively overlooked ones of the likes of referendums, federalism, electoral laws, bicameral legislatures and central banks, to name just a few. Yet the principle of popular power has no single meaning and can be articulated in a variety of different ways, which in turn offer different accounts of what makes state institutions democratically legitimate. In fact, depending on how the principle of popular power is interpreted, some institutions may appear completely legitimate, while others will not.

The history of the French Revolution offers a stark example. When debating the institutional implications of the newly established principle of popular power, representatives at the National Constituent Assembly of 1789 found themselves divided between at least two camps. Those, like Mirabeau, who understood the principle of popular power as expressed through the language of national sovereignty, believed that it could only be realised through the mediation of representatives in the assembly. By contrast, those, like Pétion, who relied on the idea of popular sovereignty tended to argue that only forms of direct or semi-direct democracy were consistent with the principle of popular power (Jaume, 1989). This divergence not only underscored different understandings of who the people were and what their political power entailed, but they also had divergent institutional implications: while supporters of national sovereignty defended the institution of the representative mandate, theorists of popular sovereignty argued in favour of the imperative mandate. The French National Assembly eventually opted for representative mandates, which became standard practice around the world. Yet the principle of popular power is still interpreted in a variety of ways which, in turn, correspond to different ways of assessing the democratic legitimacy of modern institutions. Among the various ways of conceptualising the principle according to which power belongs to the people is also the idea of constituent power, which is the subject of this Critical Exchange.

In what follows, we ask: what are the consequences of assessing the legitimacy of given institutions through the lenses offered by the notion of constituent power? To do so, we start from the premise that constituent power represents a specific interpretation of the principle of popular power. In other words, we take constituent power to be neither a term that indicates just any possible meaning of the principle, nor an abstract and indeterminate account of popular power. Instead, we believe that constituent power has a discreet meaning of its own and that, as such, it offers a specific interpretation of the principle of popular power. The specificity of constituent power derives from both its conceptual structure and its history. Starting with the former, and as the very words suggest, constituent power is part of a conceptual pair. It is a power that constitutes legal-political structures and that, as such, is in a direct conceptual relation with the idea of a constitut*ed* power. This indicates institutions as different as supreme courts and soviets, electoral laws and revolutionary assemblies. Yet they all have in common the fact that they have not constituted themselves. Instead, they owe their origins to a superior source of political legitimacy, i.e. the constituent power. Because of this intimate connection with the constituted powers, the idea of constituent power is conceptually different from other interpretations of popular power, including the more popular notion of sovereignty (Rubinelli, 2020). The latter, in all its various iterations, has no necessary conceptual connection to the institutionalisation of power. Sovereignty, by definition, is a power that is self-standing, and nothing in its conceptual structure suggests that it needs to be used to institutionalise power. Although the notion of sovereignty can of course be used to legitimise the creation of legal and political institutions, the concept itself does not bind it to a type of politics that is constituted. By contrast, constituent power, almost out of conceptual necessity, is strictly connected to institutional politics.

The specificity of constituent power as a way of thinking about popular power is also proved by the history of the idea. While different renditions of this history exist, the first modern uses of constituent power coincide with French revolutionary political thought and, more specifically, with Emmanuel Sieyès’ claim that, in 1789, the Third Estate was the bearer of the *pouvoir constituant* (Sieyès, 2014). This meant that only the productive part of society, as opposed to the nobility and the clergy, had the right to exercise political power and, through it, to constitute a new constitutional order. While arguing this case, Sieyès was careful not to confuse his own theory of constituent power with competing accounts of popular power channelled through the language of sovereignty. This was because, Sieyès maintained, the very term sovereignty entailed an absolute power that can never be restrained and that could be abused by its bearer, be it the people, the king or parliament. By contrast, constituent power is exercised only to create a constituted order, which is limited, regulated and kept in check by the fact that it is not the source of its own legitimacy and that, as such, cannot change its mandate. Starting from this first theorisation of constituent power, the idea was used widely in a variety of different contexts across the nineteenth and twentieth centuries. In each of these contexts, it offered slightly different interpretations of what it meant to say that political power belongs to the people, and it was used to legitimise widely different sets of institutional structures (Rubinelli, 2020). Yet all these different theories and uses of constituent power had one thing in common: they portrayed constituent power as being in a strict conceptual relation with the institutions of the modern constitutional state (Colón-Ríos, 2020). This relation, in turn, underscores the specificity of constituent power as a conceptualisation of the principle of popular power both from a historical and from a conceptual perspective. And it is precisely the specific relation connecting the idea of constituent power to what I have hitherto referred to as ‘institutions’ that is the subject of this Critical Exchange.

In the contributions that follow, the authors investigate the specific relation connecting the idea of constituent power to the following institutions: constitutional courts, referendums, federalism, central banks and legislatures. Some of these institutions, such as constitutional courts and legislatures, have a long historical connection to the idea of constituent power, whose features we aim to elucidate and problematize in the pages of this Critical Exchange. Other institutions, such as central banks, are not usually analysed in relation to the idea of constituent power or are mostly discussed with reference to ideas of sovereignty, as is the case of referendums and federalism. The theorists included here will take a different approach and suggest that there is much to be learnt from broadening the analysis of such institutions to include the idea of constituent power.

In the first contribution, Pasquale Pasquino examines the role of constitutional courts. He argues that courts are legitimate only insofar as they act as a ‘derivative constituent power’. This means that their role is limited to filling the gaps necessarily present in the constitution and to interpreting the principles and values informing the original decision of the constituent power. To do so, courts cannot act in the void, ignoring the specific socio-historical circumstances they are called to act upon. This means that, instead of blindly applying the values of the original constituent power, they ought to interpret them in the light of changing historical circumstances. Equally, courts cannot be captured by the executive or by the legislature and retain their legitimacy vis à vis the constituent power. On the contrary, they must maintain their independence as part of a wider system of separation of powers. It thus follows that the legitimacy of constitutional courts depends on their capacity to uphold the values expressed by the original constituent power, while integrating them with the specific interpretations of those values prevalent in a given society at a certain point in time.

Our second contributor is Joel Colón-Ríos, who asks: what are the theoretical and the practical consequences of thinking about referendums through the idea of constituent power? He argues that the very conceptual structure of the idea necessarily complicates the relationship between the referendum and democracy. If, as scholars often seem to believe, the referendum is an instance of direct democracy, then it cannot be an expression of constituent power, which is a creature of representative politics. According to the theory of constituent power, there must be a separation between popular authority and governmental power. If referendums are indeed expressions of direct democracy, then they abolish the distinction between constituent and constituted power, and transfer all powers in the hands of the people. Yet Colón-Ríos maintains that this argument is misleading. Referendums are themselves part of representative politics. They are organised according to legal procedures, often by representatives, who decide on the question to be put to the people and on the composition of the electorate. As such, the referendum is part of the constituted order, and expresses the will of the constituted powers, i.e. the representatives and the electors. It thus follows that the legitimacy of the referendum derives not from its direct appeal to the people, but from the fact that it is part of the institutional structure of the constitutional state. Yet, Colón-Ríos maintains, the constituted nature of the referendum can, at exceptional times, effectively channel the will of the constituent power. This happens only when the referendum is used to modify the constitution or to establish a mandate to create a new constitutional text. In these rare occasions, the referendum obtains legitimacy, not from the constituted order, but because of the constituent will it expresses.

In our third contribution, Eva Marlene Hausteiner analyses the role of constituent power in federal states. This question, she argues, demands special attention because of the peculiarly complex nature of federal constitutions. These are indeed prone to radical change, which takes place outside the limits imposed by the constitution but does not overthrow it. Good examples are processes of annexation and secession, or changes in the distribution of power across regional, state and federal levels. These changes are the expression of neither a constituent power of revision, as they are not regulated by the constitution, nor of a fully-fledged constituent power, as they do not create a new constitutional order. By contrast, Hausteiner suggests, we should consider them the expression of a *re-constituent* power. Further, the subject of constituent power is also different in federal states. While the source of legitimacy of unitary constitutions is identified in the will of a ‘unitary people’ – as fictional as this unity effectively is – federal states are made of a multi-layered *demos*. This means that the subject of constituent power expresses itself through a multi-layered process of decision-making which, Hausteiner suggests, adds up to a more demanding standard of democratic legitimacy and could be called *pouvoir constituant mixte*.

Hjalte Lokdam’s contribution to this Exchange questions whether central banks in general, and the European Central Bank in particular, are to be considered democratically legitimate. He argues that the idea of constituent power, although only rarely applied to central banks, can offer a valuable frame to answer this question. First, Lokdam maintains that, differently from other central banks, the ECB could effectively be thought of as a product of the European constituent power, although this should be seen as a multi-layered power acting through extraordinary representatives in a federal setting. Second, Lokdam asks what are the implications of thinking about the ECB through the idea of constituent power. While, on the one hand, associating the ECB to constituent power bestows it with democratic legitimacy, on the other hand it engenders some risks. This is because, if the mandate of the ECB comes directly from the people, then it must be a rigid mandate, which cannot be easily bent to changing political circumstances. Yet, in moments of crisis, such as the sovereign debt crisis of 2011, this very rigidity might become an obstacle to prompt and swift action and result in the suspension of the ECB’s democratic mandate. This, in turn, gives a free hand to the unelected experts and insulates their acts from democratic contestation. Whether a European constituent power could give a more flexible mandate, and hence bestow both legitimacy and flexibility on the ECB, remains an open question.

Our final contribution is by William Selinger. He asks how the role of the legislature would change if we assessed it in relation to the idea of constituent power. At first sight, Selinger suggests, it looks like the power of the legislature would have to be limited by the fact of being a constituted power. This argument is further strengthened by the fact that circumscribing the power of the representative assembly was one of Sieyès’ main goals when theorising constituent power. Yet, Selinger argues, if we stopped thinking about the legitimacy of legislatures in terms of sovereignty, and instead started referring to constituent power, we might come to a surprising conclusion. The idea of constituent power, when associated to legislatures, strengthens their power and that of the representatives who sit in them. This is because constituent power depicts the people as the original source of power, which, however, disappears in the background during times of ordinary politics. It thus follows that, differently from what happens with the idea of sovereignty, the people cannot be considered the ultimate decision-makers. On the contrary, this power is vested in the representatives, who nonetheless are a constituted power and thus have to act within the limits of their constitutional mandate. In other words, Selinger maintains that thinking about legislatures through the lenses of constituent power both limits and strengthens their power: it limits legislatures because it clarifies their constituted nature, but it strengthens legislatures because it insulates them from the idea of an overarching sovereign will of the people.

The aim of this Critical Exchange is to underline the conceptual and historical specificity of the idea of constituent power. Far from being just a synonym for sovereignty, it offers a specific way of interpreting the principle of popular power, one that ties its exercise to the fundamental institutions of the modern state. And when assessing their legitimacy through the idea of constituent power, it becomes clear that some institutions are, as a result, strengthened in their relationship to the principle of popular power (constitutional courts and legislatures), while others invite a thorough questioning of their function within the constitutional state (referendums, federalism and central banks). This proves that there is much to be gained from thinking about democratic politics through the idea of constituent power. Not only does it demand that we distinguish between different ways of conceptualising the principle of popular power; it also forces us to clarify how we assign democratic legitimacy to our institutions and why.

Lucia Rubinelli

## Constitutional courts and amending constituent power

Constitutions, more specifically written liberal constitutions – the only ones I’m going to consider in the following remarks – are a set of rigid, entrenched legal norms concerning the structure of the government and the fundamental rights it must guarantee to the members of a political community. The well known exceptions in this family are the customary constitution of the United Kingdom and the mix of written but flexible laws and unwritten conventions making up the constitution of New Zealand. Rigidity refers to the existence of complex legal procedures (more demanding than those used to enact ordinary laws and often super-majoritarian) to modify the constitutional status quo, procedures, that include either at least part of the parliamentary opposition or more or less direct popular decisions (Albert, 2014; Albert, 2019; Report on Constitutional Amendment, 2009; Ehmke, 1953). It is important to emphasize that rigidity is no more than a legal quality of the fundamental laws of a country, and it is not, as such, equivalent to the stability of a constitutional order. The latter depends on concrete specific historical, political and economic circumstances, so it is not surprising, given the political history of the country from the Revolution onward, that France had many more rigid constitutions since 1791 than the United Kingdom, which instead slowly modified over time its flexible customary constitutional order (Bryce, 1901, pp. 124–213; Lasalle, 1862).

It is even more relevant, given my focus, to distinguish the constituent power *stricto sensu* from the power to amend and modify the constitution – the French doctrine speaks of original and derivative constituent power. The theory of constituent power, introduced in the continental European debate, notably by Sieyes and further developed by Carl Schmitt in his *Theory of Constitution* (1928), refers to a foundational moment, which marks a radical break with a previously existing (mostly colonial or monarchical) legal order. In liberal democratic representative regimes, constituent power entails some form of popular participation in the process of ratifying and enacting the new fundamental order of the political community delineated in the constitutional document. Moreover, constituent power supports it over time.

The *pouvoir constituant dérivé* is instead a constituted power, typically specified in the written rigid constitution, a concept which has rarely been the object of systematic investigation (Levinson, 1995). As said, it is normally exercised either by a supermajority of the elected representatives, or by a mix of representative and popular decision-making, sometimes via referendum. Additionally, constitutional courts, in their own way, exercise some form of constitution amending power (for the South African exception, see Gloppen, 2018). This is not the original constituent power in its radical version: courts modify the constitutional order by filling its gaps or by interpreting its principles and values, and I shall try to explain briefly why and how.

To understand such unusual claim, i.e. that courts exercise a derived constituent power, we need to be aware that all written constitutions are inevitably ‘incomplete contracts’. Even the best and less short-sighted founding fathers cannot foresee all the possible questions, cases and controversies that can rise under the constitution they write. This is particularly true when the constitutional document is old and survived for a very long time, as in the American case. So, saying that a constitutional court is just ‘enforcing’ the constitution, when a case or a question emerges and the court is asked to adjudicate it, does not make much sense. It is implausible to argue that interpreting the constitution means simply looking back at the ‘original intent’ of the founders. It is a strange exercise to speculate about what the founders would have thought about questions unimaginable at the end of the eighteenth century (speaking of the US constitution), as is evident in the case of what should be done about internet regulation, or same sex marriage or Covid-19.

Instead, I will argue that constitutional courts act as derivative constituent powers by presenting two examples drawn from much more recent constitutions. The first has to do with the Italian republican charter enacted in 1948, the second with the French constitution of the Fifth Republic.

Among the powers that the constitution assigns to the Italian constitutional court there is the adjudication of the so called *conflitti di attribuzione* (conflicts of institutional competences) among high state organs – notably the legislative, the executive and the judiciary, or between the national government and the regions (see art. 134)*.* A similar competence exists in the German (*Organstreitigkeit*, art. 93) and the Spanish constitutions (art. 161 c.). The Court is thus given the important task of defending the structure of divided power of any liberal constitution. When a conflict between the organs of a pluralistic authority emerges, the system needs a judge to avoid the derailing of the anti-monocratic authority established by the fundamental law.

On 19 October 1995 the Italian Senate voted a motion of non-confidence against the attorney general Filippo Mancuso, who sued the upper house before the constitutional court for the violation of art. 94 of the Italian constitution, which regulates the process of the government’s investiture as a single body. Mancuso argued that the constitution speaks of a vote of confidence and no-confidence concerning the government as a collegial body and that a no-confidence vote against a single member of the government was unconstitutional, so that, in his opinion, the entire government should have resigned. In its decision of 18 January 1996 (Sentenza Corte Costituzionale 7, 1996), the court rejected the interpretation of the constitution presented by Mancuso and declared that the articles of the constitution concerning the government’s political accountability vis-à-vis the houses of the parliament did not exclude the vote of no-confidence addressed to a single member of the cabinet.

In such cases, it is unrealistic to claim that the court is merely implementing the constitution. Through an interpretation of constitutional articles concerning executive accountability (based on the history of parliamentarism in other European countries – even though this research is not mentioned explicitly in the court’s opinion), the judges were filling a gap in the constitutional text. In fact, they wrote a little extension of the fundamental law, responding or supplementing the existent blindspot. In a limited, marginal sense they were amending or integrating the constitution – something that neither the parliament nor the minister would have been able to do, because an actor cannot be a judge in their own trial, without destroying the structure of divided power in liberal constitutions, and thereby establishing a monocratic sovereign state organ.

It may be noticed that even in the UK, where there is no written and rigid constitution, a court of justice – the supreme court – was recently asked to decide on a conflict between parliament and the prime minister, and not surprisingly it decided in favor of the consolidated doctrine of parliamentary sovereignty. This judgement shows that the sovereignty in question is not so absolute as to exclude a conflict between the parliament and the prime minister that may emerge and thus will be judged by an independent judicial body (Miller (Appellant) v The Prime Minister (Respondent), 2019).

The second example supporting my claim is the decision of the French *Conseil constitutionnel* of 15 January 1975 concerning the statute on *interruption volontaire de grossesse* (abortion) voted by the parliament but referred before its promulgation to the council by a minority of representatives hostile to it.^1^

The hybrid nature of the French constitutional council*,* which used to be a mix between a court of justice (as it largely is now) and a sort of executive organ of the constitutional system (as was the case in 1958) is the origin of a unique practice: the internal deliberations among the members of the council are recorded, and, as of 2008 the proceedings are publicly available – since the required 25 years have lapsed. The court decided to uphold the *loi* Veil, which legalized abortion (Mathieu et al., 2009, pp. 266–286). The decision and the debate in the council are of special interest for a variety of reasons, among others the fact that in 1975, no member of the council was expecting that the transcript of the debate would have been made public. Here I shall focus only on the arguments of the *juge rapporteur* François Goguel, which was accepted by the council. Goguel, a Catholic believer, declared himself to be personally against the norm approved by parliament, but he nonetheless admitted to seeing no obstacle in the constitutional text as to the constitutionality of the statute, notably because the constitution did not say anything about the question.^2^ Here again it seems that what is not forbidden by the fundamental law may be considered compatible with the hierarchy of norms it enshrines, provided that the court agrees on that compatibility. Therefore we can safely say that in this case the constitution was integrated and expanded by the decisions of the court.

The constituent power of the courts is thus mostly based on a process of integration of the constitutional text. This integration is marginal, because there needs to be no strong opposition in public opinion and/or among the elected representatives. It thus follows that the type of amending power that lies in the hands of constitutional courts’ judges is incremental. The scope of this power depends largely on the cultural and political circumstances of each given society. By their decisions, courts may be able to simultaneously preserve and refine the constitutional structure of a liberal regime and to enlarge the understanding of the rights it is meant to guarantee. In that sense, they cannot change the basic structure of the constitution, but they can marginally interpret and rewrite its content – for as long as they keep their relative independence vis-à-vis the political (democratically elected) actors.

Courts are certainly not all-powerful institutions. They only work thanks to the support of public opinion and acceptance by the elected representatives. As a counterexample, one can think of the constitutional courts of Hungary and Poland captured by the executive after the illiberal turn of those regimes, whose legitimacy is seriously contested. In liberal societies, based on the principle of divided power and its polyarchic structure, constitutional courts are actors exercising a limited, incremental derivative constitution-rewriting power.

Pasquale Pasquino

## Constituent power and referendums

When the idea of constituent power is deployed in contemporary constitutional discourse, it is often associated with referendums. This is hardly surprising, for in contemporary societies, referendums are the key mechanism for the formal involvement of the citizenry in the making of political decisions. Upon closer examination, however, the connections between referendums and constituent power are much more tenuous than what may otherwise appear. This is not to deny that the prevailing view rests on apparently strong grounds. On the one hand, referendums are historically related to the imperative mandate, and in the eighteenth and early nineteenth centuries, the imperative mandate was frequently seen as the means through which the constituent subject controlled the actions of those sitting in the constitution-making assemblies. On the other hand, although the theory of constituent power presupposes a representative form of government (i.e. a distinction between constituent and constituted authority), the democratic exercise of constituent power seems to require the type of direct popular involvement facilitated by a referendum. This contribution explores the connections and disconnections between constituent power and referendums from a theoretical perspective and from the perspective of actual constitutional practice. On the theoretical front, it argues that while referendums cannot exhaust the nature of constituent activity, they can play an important role in the exercise of constituent power. The practical implications of this view, my contribution shows, have been exemplified in the jurisprudence of the Colombian Constitutional Court.

Referendums are the main formal mechanism of direct democracy in societies whose size makes it impossible for the entire citizenry to assemble in a single law-making body. In theory, they allow the electorate to make decisions about specific political issues and, in many if not most constitutional arrangements, those decisions are binding on all representative and governmental institutions. The institutionalisation of the referendums is, in this sense, a democratic correction to representative democracy which, apart from the episodical vote to elect representatives, does not require any form of direct popular intervention in the making of political decisions. Referendums are typically (though not exclusively) reserved to moments of constitutional change, times where the electorate is asked to authorise a modification of the fundamental laws of the state. It is in those moments, and not during the adoption of ordinary laws or policies, that democratic principles seem to demand direct citizen involvement. If constituent power is understood as the popular power to create and change constitutions, and if referendums allow the electorate to ratify or reject a draft constitution or a proposed change to an existing one, then they seem to be the obvious mode of exercising constituent power in contemporary constitutional orders.

That is how the relationship between constituent power and referendums is generally understood by constitutional lawyers. This approach assumes, wrongly in my view, that the ideal institutionalisation of the exercise of constituent power would be direct democracy. The theory of constituent power is in fact a creature of political representation: in a direct democracy, a system where all laws, including the fundamental ones, can be drafted and adopted by the entire citizenry, there is no need for the theory of constituent power, which is a theory about a separation between constituent (popular) and constituted (governmental) authority. Accordingly, if referendums are to be understood as a possible institutionalisation of constituent activity, the fact that they are a formal mechanism of direct democracy is not enough. Indeed, there may be other mechanisms that, because of their deliberative, participatory, or inclusive nature, may be more appropriate for the exercise of constituent power than a simple yes or no vote in a referendum (In the second part of my contribution I consider the reasons why the traditional view, despite overstating the connections between constituent power and direct democracy, is nonetheless right in attributing to referendums a key role in constituent activity).

Now, like constituent power, the institution of the referendum is also a creature of political representation. The *raison d’être* of referendums is the need to submit to the citizenry certain decisions that, because of their constitutional significance, should not be left solely in the hands of representatives. Seen from this perspective, referendums play a similar role to that assumed by the imperative mandate at different points long before the great revolutions of the eighteenth century. Before its almost universal abolition, the imperative mandate served as an important link between representatives and the voters who elected them. The idea that representatives are bound by citizens’ instructions, although in theory applicable to every kind of decision, usually acquired a special importance in the context of constitutional change, that is, during the exercise of constituent power (Wood, 1998, p. 191). The imperative mandate appeared, in the eyes of some, as the means through which the citizens who sat in primary assemblies and town meetings could control and influence the conduct of delegates called to engage in constituent activity. While the abolition of the imperative mandate meant that citizens could not influence the actions of those delegates *ex ante*, the referendum allowed them to control them *ex post*. In fact, as Pedro de Vega has shown, in the Middle Ages, the word ‘referendum’ was used to refer to communications between delegates and their electors about issues that emerged before the assembly and that had not been specifically included in the former’s mandates. Delegates would express opinions on those issues, *ad referendum*, that is, subject to the subsequent ratification of their constituents (1985). Despite these connections, the difference between the imperative mandate and the referendum (the first one taking the form of an *ex ante* instruction; the second one of a potential veto) has important implications in the context of constitutional change in current legal systems.

Consider the participation of the electorate in constitutional reform, one of the instances in which contemporary constitutions tend to require the direct participation of the electorate. In the context of constitutional reform, the electorate does not act as a constitution-*maker* but as an institution of control. It does not create new constitutional forms or is even necessarily consulted about what those forms should be, but rather vetoes or confirms decisions about constitutional content made by others. Those others could take the form of an elected constitution-making body, of a legislature acting through a special majority, of an ordinary legislature, or even of a non-elected commission of experts. Moreover, the referendum will be subject to a set of legal procedures that limit in important ways the extent of popular participation (e.g. a yes or no vote, no formal deliberation), that may only identify as voters those individuals that had previously met certain more or less arbitrary eligibility criteria (e.g. criteria about age, residency), or that may give more weight to the votes of the minority (as any decision-rule other than 50%+1 would do). Those kinds of procedural limits seem more consistent with an electorate playing the function of a state organ, of a constituted authority, than with a popular exercise of constituent power. The question then becomes whether the electorate, when it acts through a referendum, could ever be understood as exercising constituent power. I now turn to consider a possible answer to that question. Can the electorate be understood as more than a state organ playing a discreet function within a larger process of constitutional reform? Can it be (or should be) seen as a juridical manifestation of the constituent people (Colón-Ríos, 2020)? Some constitutional theorists have explicitly considered these questions. Carl Schmitt, for example, maintained that ‘even the constitutional powers and competencies of the “people”, which is to say the state citizens entitled to vote’ (such as the referendum and the initiative under Articles 73 and 76 of the Weimar Constitution), are not ‘powers of the sovereign people, who give themselves a constitution and engage in acts of the constitution making power. They are, rather, competencies in the context of the constitution that is already provided’ (Schmitt, 2008, pp. 145–146). For Schmitt, to the extent that a referendum only allows the electorate to act according to a legally controlled process of constitutional reform, it cannot be a means for the exercise of constituent power. According to Schmitt’s understanding of the limits of the power of constitutional reform, this means that a constitutionally regulated referendum will not be enough for the legitimate alteration of the *material constitution* (i.e. the constitution’s fundamental content, which Schmitt called the ‘substance of the constitution’), something that falls under the exclusive jurisdiction of the constituent subject and that cannot be subject to determinate constitutional procedures (Schmitt, 2008, pp. 77–79).

This view has been embraced by the Colombian Constitutional Court in a series of judgments. A democracy, the court has maintained, cannot be participatory unless the people can also appear as the bearer of the power of constitutional reform (Schmitt, 2008, pp. 77–79). In Colombia, this was facilitated through what the court identified as the ‘constitutional referendum’ regulated by Articles 377 and 378 of the Constitution of 1991. But the court made sure to point out that the inclusion of the referendum as part of the mechanism of constitutional reform was not equivalent to the establishment of a ‘pure direct democracy, not subject to judicial control’ (Judgment, C-551/03 (n 49) para 44). ‘The power of constitutional reform, even when it includes a referendum’, the court stated, ‘is the deed of neither the originary constituent power nor of the sovereign people, but an expression of a juridical competency organised by the Constitution itself’ (Judgment, C-551/03 (n 49) para 40). For that reason, the court maintained, such a power is always limited by the impossibility of replacing the constitution. Otherwise, the power of constitutional reform would become equivalent to the originary constituent power (Judgment, C-551/03 (n 49) para 40; the French Constitutional Council rejected this kind of approach in its decision no 62-20 DC, 6 November 1962). Some years later, the same court made this point even more expressly: ‘The referendum as a mechanism of constitutional reform is always a manifestation of the derived constituent power [i.e. the limited power of constitutional reform that has been delegated to the ordinary institutions of government] and not even the intervention of the electorate…has sufficient juridical force to transform a referendum into a foundational, primary, or originary constituent act’ (Judgment C-141/10, Colombian Constitutional Court, para 1.3).

The type of electoral acts examined in those judgments can be identified, as suggested by the court itself, as *constitutional referendums*. That is, referendums through which a proposed constitutional change is approved or rejected as part of a procedure established by the constitutional amendment rule. But not all referendums are like that. Indeed, the Colombian Constitutional Court has distinguished between situations where the people, acting ‘outside of any normative channel, decides to alter the constitution or give itself a new one’ and situations ‘where the citizenry acts as a constituted organ, and accordingly, as a limited one’ (Judgment C-140/10, Colombian Constitutional Court, para 1.4). In the former scenario, the people act as the constituent power and, in the latter, it operates as a constituted one (Judgment C-180/07, Colombian Constitutional Court, para 2.2.2.2.1). Importantly, the court included within the latter type of situations those cases in which, ‘according to constitutional provisions, the people is convened to decide whether to call a national constituent assembly’ ((Judgment C-180/07, Colombian Constitutional Court, para 2.2.2.2.1). That view is problematic, because such a referendum would be authorising an entity to replace the existing constitution or to alter its material content. The fact that it is convened according to law does not seem to be a sufficient reason for depriving it of a constituent nature.

As Ernst-Wolfgang Böckenförde stated, it is true that there can be a juridical distinction between ‘the people as an organ and the people as sovereign; however, the two cannot be separated as though they were two distinct and real entities: in the final analysis, they are the same “people”’ (2017, p. 179). ‘[A]nytime the people takes an active role as an organized entity’, he added, ‘the unorganised people of the *pouvoir constituant* is also involved and present in some way’ (2017, p. 179).

Authorising and ratificatory referendums, as long as they involve the alteration of the material constitution, should be understood as *constituent referendums*, i.e. instances in which the people exercise their constituent power directly. In the case of a referendum calling for a constituent assembly authorised to adopt a new constitution (within or outside the established amendment rule), the electorate could be understood as commissioning that entity with the production of material constitutional content. That is, an instance in which ‘the people’ acts through the electorate and may set conditions to which a constitution-making body will be subject. Such conditions may include the creation of a specific type of constitutional content within a certain period. They may also require the submission of the new constitutional text to popular ratification. In that final referendum, the people, acting once again through the electorate, not only accepts or rejects a draft constitution, but also confirms that its mandate has been respected.

To conclude, I hope to have shown that the relationship between constituent power and referendums is not as clear as it is sometimes thought. While there are important affinities between the institution of the referendum and the exercise of the power to create new constitutions, there are also significant tensions between them. These tensions emerge from the fact that a referendum will normally be regulated by a set of norms potentially inconsistent with the exercise of a truly popular constitution-making jurisdiction. Simultaneously, however, a referendum provides a means for the entire electorate to participate in the authorisation of a constitution-making episode, as well as in the ratification or rejection of important constitutional changes. In such a context, I argued, referendums can be understood as a key part of the exercise of constituent power in contemporary societies, when a people, acting through the electorate, participates directly in the alteration of their country’s material constitution both by issuing a mandate for the creation of new constitutional content and by determining if such a mandate has been respected.

Joel I. Colón-Ríos

## (Re-)Constituent power and federal change

The concept of constituent power revolves – not least due to its prominent role in French revolutionary thought – around at least two focal points, which endure in spite of the idea’s highly contested history (Rubinelli, 2020). On the one hand, constituent power is still frequently associated with political founding; on the other, many theorists continue to tie it to questions of popular sovereignty in unitary democracies. These emphases in the conceptualization of constituent power, however, divert attention away from constellations both more complicated and more common than the founding of unitary states.

The locus and legitimacy of constituent power should be examined not only with regard to the moment of founding. Political orders can also substantially transform after their foundation in ways not projected by the founders and yet without undergoing revolution. This raises the question of who authorizes and directs such changes: Where does *re-*constituent power, understood as political power effecting non-revolutionary but radical transformation beyond constitutional confine, reside after the founding? I define re-constituent power – as I will elaborate further below – as different from amending power or *pouvoir constituant constitué*, in that its transformative power does not operate within the rules of the constitution, including the rules for constitutional amendment. Re-constituent power is thus related to what Yaniv Roznai has called the sporadic ‘re-emergence of the primary constituent power’ (2017, p. 25) – but it emphasizes the potentially new configuration of the constituent subject in a situation of re-constitution.

With regard to the second conceptual limitation, constituent power has been closely connected to the idea of a state constituted through the will of *one* demos – even if this demos is potentially pluralist. But many modern democracies are constitutionally structured in political layers, resting on the political fusion and co-operation of multiple citizenries: they are federal, and as such they not only distribute power through a complex institutional structure. Moreover, this structure is often assumed to have been originally constituted by multiple collective actors. Federal polities consist of different spheres of rules, which significantly complicates the sharing and exertion of power – even when the constituting populations are not thought of as separate peoples, let alone nations. This layering and sharing of power among multiple collectives also affects (re-)constituent power.

The dynamics of deep transformation engrained in federations concern both these aspects of constituent power – its continued importance in established institutional settings, and its complications in heterogeneous polities – and they raise questions that have so far remained unresolved: How can (re-)constituent power be located in federal political orders? And how does this power relate to the territorial and political segmentation of the demos into sub-units, with both the federal and the regional level involved in the democratic process?

From the vantage point of these questions, federal polities are a particularly interesting and difficult area for identifying the locus and essence of constituent power, because here the ‘paradoxes’ of constituent power (Loughlin and Walker, 2018) as the power to (re)constitute a political order, are intensified. Does the power constitutive of federations coincide with the power that transforms them? Does, therefore, the founding constituent power constitute an ongoing constituent power? The main interest in the following lies less in gauging the conceptual depths of constituent power through the lens of federalism, but rather in asking: what can the lens of constituent power contribute to our understanding of the complicated functioning and transformation of federal democracies?

Carl Schmitt, whose emphasis on political decision with regard to constituent power is well-known, saw the fate of federal political orders as largely outside the control of political decision-makers. Federations, and particularly federal democracies, were bound to move almost automatically towards homogeneity – towards centralization up to the point of unitary statehood (Schmitt, 1928, pp. 363–391).^3^ Even if we question this thesis of a centralizing magnetism, Schmitt’s view succinctly highlights the dynamic character of federalism. Federalism, as an institutional framework, may carry the promise of stability and balance under often tumultuous circumstances, and federal constitutions are frequently marked by a high degree of rigidity – but most federations regularly undergo changes and conflictual dynamics of varying magnitude.

Some of these dynamics are regular and expected elements of the political and constitution-amending process – for instance the drawn-out negotiations and compromises between levels of government and their respective executives and legislatures, or the re-setting of boundaries on competences, for which oftentimes constitutional court decisions are necessary (Bednar et al., 2010). These balancing acts within a multi-layered, complex polity have been extensively analyzed (e. g. Benz and Broschek, 2013).

But federations are prone to much more fundamental transformations, which raise the question of constituent power. Discussing radical centralization, Schmitt has named just one of the directions such transformation processes can take. The shift from politically decentralized to centralized statehood can – depending on the political system – occur through legislative or judicial routes but also through more informal redistributive effects. But federations also frequently expand or contract in terms of membership. Finally, what has been called ‘federal failure’ can also be regarded more neutrally as disintegration (Franck, 1968; Patberg, 2019) – a process requiring, as other types of deep federal transformation, collective authorization in order to be democratically legitimated. All these types of transformation can be considered changes to the polity’s essence, in the sense that they shift its composition, power distribution, and political rules.

Although in some existing federations, the modalities for federal transformation are prescribed by the constitution (Aroney, 2017), only very few countries regulate all the types of deep and lasting transformation.^4^ In Germany, for instance, the overall federal structure is an inalterable element of the constitution,^5^ but the competences of both federal centre and states can be – and have been – rearranged through constitutional amendment; as for the accession of new member states, the Basic Law until 1990 contained only an enlargement provision. Article 23 (1949) was supplanted by the so-called ‘Europe-article’, which allows for the transfer of competences to the supranational level – itself a provision for a different federation-transforming act. Similar, though much more elaborated, procedures exist in the EU’s treaty framework – but here as in many other federal contexts, both regulated and unregulated modes of federal transformation co-exist. In the EU, unregulated centralization over time towards a tightly integrated federal order on the one hand, as criticized by Weile (1991), is accompanied by a tightly regulated accession process on the other (cf. Schimmelfennig and Sedelmaier, 2002). In the United States, the process of increasing centralization has been continuing almost since the founding – propelled not only by Supreme Court decisions, but also resource redistribution, population movement to the coasts, and, of course, the Civil War.

Surprisingly often, therefore, fundamental changes in the essence of a federation are not provided for in its constitution, but take place nonetheless. This practice of informal or unregulated transformation concerns not only drastic centralization but also changes in territory and the number of sub-units. Not only do some federal constitutions not provide for enlargement: very few provide for secession or expulsion – a fact that does not prevent these transformations, as the example of the separation of Singapore from the Malaysian Federation in 1965 shows (Hausteiner, 2018).

It is here that a key tension becomes apparent. Federations are oftentimes designed to ensure stability under conditions of diversity, conflict, and strong centrifugal forces, and this desire for stability becomes manifest in a certain degree of constitutional rigidity (Aroney, 2017, p. 7). This very rigidity, however, clashes with a key characteristic of federalism – its dynamic character. This dynamism arises out of the multiplicity of territorially entrenched collective actors and their political goals. Federal arrangements may be founded in the hopes of avoiding fundamental re-constitution, but the conflicts around power and particularly around the terms of membership – both between federal and regional levels and among the sub-units – tend to build pressure toward re-arrangement. This characteristically federal dynamism can amount to a fundamental restructuring of the overall polity: its extent, its governing rules, and its self-conception.

Since federations are characterized by this tendency towards deep structural transformation, the question arises of the nature of constituent power within established federations. How is constituent power embodied in federal states – what constitutes it, who exerts it, and when can it be deemed democratically legitimate? Here, three points are of particular relevance.

Firstly, any consideration of constituent power in federations needs to determine the threshold beyond which federal transformation amounts to re-constituent action. Surely, constitutional amendments – as inbuilt and pre-regulated elements of the political system – are below this threshold of constituent power. Even if they produce fundamental changes – such as an increase of competences for the federal centre or new rules for the admission of new members to the federation – they are not operating outside the existing political rulebook but are part of the amending power or *pouvoir constituant constitué* provided for by the constitutional framers.

However, if federations undergo transformations short of full-fledged revolution yet fundamental and outside the bounds of the constituted order, it is plausible to speak of re-constituent action. As explained earlier, due to efforts for federal stability, many constitutions do not provide for the eventualities of expansion, secession, expulsion – thus leaving room for the not-too-unlikely case of extra-constitutional transformation. If these eventualities of federal re-arrangement are not covered by the constitution, they alter the constitutionally established order. Importantly, even these transformations do not operate wholly outside the existing rulebook, but are to some extent pre-structured by it. In the case of Singapore’s expulsion from Malaysia, for instance, both the federal parliament and the federally elected (though mainland-dominated) executive claimed the authority to move against the recalcitrant member state. Even if re-constituent power therefore is not simply a pre-constituted amending power, it is related to the powers constituted through federal foundation: the latter creates the political preconditions for further transformative action.^6^

This leads to a second point: the characteristically non-monolithic nature of constituent action in federal constellations. Here the link between constituent and re-constituent power is of particular relevance. Especially in what Alfred Stepan has called the ‘coming-together’-type of federation (Stepan, 1999) – a federation formed through a joining of several political entities – the federal structure itself is a result of collective decision-making among multiple polities. For understandings of constituent power which emphasize its close connection with popular sovereignty, the split character of the federal *demos* is especially relevant. This is due to the internally pluralist structure of constituent action. In a polity defining itself through territorial segmentation, the likelihood that re-constituent action – be it direct popular action or action relying on popular approval – emanates from complex coalitions is considerable; and this complexity can grow after the federal founding, if multi-level pluralism is combined with the pluralism of democratic society. This diagnosis of twofold pluralism runs directly counter Schmitt’s description of federal democracy, the key characteristic of which is an inevitable tendency towards homogeneity. (Re-)Constituent power in democratic federations is thus shaped by its multi-layered and pluralist environment. This also means that there is a particularly high threshold for constituent action to be democratically legitimate, since a plurality of groups and actors must be considered in unstructured processes of deep transformation. Centralization, expansion, or expulsion at the initiative of actors from only one sub-unit, or from only the federal level, would hardly satisfy the legitimacy requirements associated with popular sovereignty.

Finally, this high legitimating threshold at the same time holds the promise of a particularly substantial legitimacy of (re-)constituent power. As Peter Niesen has argued with regard to the federal structure of Europe, the mechanism of a *pouvoir constituant mixte* – a term applied by Jürgen Habermas to the case of the EU^7^ –, in which each citizen exerts power both through her member state *and* the European parliament, facilitates supranational legitimacy. This theorization of mixed constituent power is, according to Niesen, not primarily concerned with constitutional transition, but it considers its operation beyond the foundational moment and potentially throughout the existence of the federal arrangement (2016, p. 220).

Additionally, more radically transformative change, situated in the space between the founding and revolutionary politics, can gain particularly substantive legitimacy thanks to the peculiar structure of federalism. Ideally, federal structures are not only able to secure civic participation and accountability on multiple levels: they also prepare citizens to consider more than one political arena for engaging in transformative political action. (Re)constituent power in federal constellations can therefore draw from more than one source of democratic legitimacy, due to the dual role of each citizen as a citizen both of a sub-unit and of the federal polity – a role firmly defined for ‘regular’ political processes through the constitution, but also relevant for moments of federal re-ordering.

For federations, therefore, the answer to the question of legitimate substantive change outside of formal constitutional provisions is particularly significant because of their tendency toward such transformations – and because of the pluralist nature of democratic authority entrenched in their structure. The language of constituent power can illuminate these dynamics if it eases its focus on political founding as well as on the territorial unity of the demos – and takes into account the complicated ways in which the original constituent power shapes later constituent action, without firmly predetermining it. As federal histories show, pathbreaking foundational moments can, in a risky balancing act, both stabilize a polity and leave open significant space for political change.

Eva Marlene Hausteiner

## The central bank and the constituent power

Can central banks be institutions of the constituent power? If so, what does this mean in terms of their political role and their legitimacy? In tandem with the increasing involvement of politically independent central banks in governing our economic lives, the question of their democratic foundations and legitimacy arises. This is because central bank legitimacy fits uneasily within the mechanisms of ordinary democratic politics. Elections are rarely, if ever, decided on questions of monetary policy, and it is often unclear what power, if any, elected politicians have over central banks. The question of the relationship between the central bank and ‘the people’ is therefore of some importance.

This does not have to involve the question of the constituent power, but it may. With reference to the European Central Bank (ECB) – one of the few central banks in the world that can meaningfully, albeit controversially, be considered an institution of the constituent power – this contribution discusses the consequences of thinking about central banks through the lens of constituent power. While reference to the constituent power promises to establish a firm democratic foundation for the central bank, the elevation of the central bank’s mandate and authority to the constitutional level comes with certain problems. In particular, it risks rendering the mandate of the central bank too rigid to be practical in crises, thereby prompting a politics of suspending or altering the mandate in an emergency situation. This, of course, is often anything but democratic.

Most central banks are not institutions of the constituent power. Their position within the modern state has developed gradually and been entirely elite-driven. They are constituted powers, of course, but their authority is derived from other constituted powers that can withdraw or alter them at will. They are products of secondary law, not the primary law of the constitution, and were created by ordinary political representatives working within constituted legislatures, not ‘the people/nation’ or its extraordinary representatives in revolutions or constituent assemblies. As such they could be called ‘secondary’ constituted powers as opposed to the ‘primary’ constituted powers that create them (typically legislatures).

Some central banks, however, have been created in extraordinary political moments. The post-World War II German central bank, for instance, was created a year before the Basic Law constituted the Federal Republic in 1949, and it enjoyed an extraordinary position in the life of the West German state. Following its creation, the Bundesbank quickly became a symbol of a break with Germany’s past and presented itself as a bulwark against the dangerous excesses of politics. Through actively cultivating public opinion in its favour, the Bundesbank successfully established itself as an independent power within the state on a par with the legislature and government. In conflicts with the government, the Bundesbank appealed to ‘the people’, and, more often than not, it carried the day (Mee, 2019). The Bundesbank, however, was formally still a secondary constituted power, as the Bundestag held the right to alter or abolish it through ordinary legislation. If there was a connection between the German people and the Bundesbank, it was informal – but no less effective for that.

When the European Central Bank was created, the Bundesbank was the main source of inspiration. Like the Bundesbank, the ECB was created in a moment that marked a transformational break with the past. Like the Bundesbank, the ECB was to be independent of political instruction. Unlike the Bundesbank, however, the ECB’s mandate and institutional status were fixed in primary law, the Maastricht Treaty. This means that no constituted power has the right to alter or abolish the ECB through ordinary legislation. Its acts cannot be vetoed, and it cannot, in principle, be compelled to do anything against its will. In matters pertaining to its Treaty mandate, it can legislate without the involvement of other constituted bodies and execute its will throughout the territory of the Eurozone without the involvement of Member State authorities. The only check on the ECB’s powers is judicial: it must act in accordance with the mandate given to it by the Treaty.

Such powers, combined with the independence from political authorities, make the ECB unique among central banks. The question is whether it is an institution of the constituent power.

The answer to this question depends to a certain extent on how the constituent power is conceptualised. In *Dictatorship,* Carl Schmitt (2014, p. 123), referring to Sieyès, defines the constituent power as

the primordial force of any state … From the infinite, incomprehensible abyss of the force [*Macht*] of the *pouvoir constituant*, new forms emerge incessantly, which it can destroy at any time and in which its power is never limited for good. It can will arbitrarily. The content of the willing has always the same legal value like the content of a constitutional definition.

In this definition, there is nothing inherent in the idea of the constituent power that precludes the possibility of central banks being institutions of the constituent power. Nothing, after all, can prevent the nation/people from creating whatever constitutional forms it desires. Thus, while central banks are not ordinarily institutions of the constituent power, it is a theoretical possibility.

Possibility, of course, is not actuality, and there are some difficulties associated with the concept of the constituent power in the context of the European Union. One of these is that the treaties were not products of constituent assemblies (except the failed Constitutional Treaty), but rather intergovernmental conferences. The primary law of the EU is thus not a product of a formless constituent power, but of an agreement between several constituted powers. Again, however, the theory of the constituent power, as formulated by the Abbé Sieyès (2014, p. 91), can allow for this through the concept of extraordinary representation. What distinguishes the adoption of the EU treaties from other international treaties is that it transforms the political status of the signatories – ‘from nation states to Member States’ (Bickerton, p. 2012) – as well as how they govern themselves (Larsen, 2021). In the Eurozone this is particularly clear, as the creation of the ECB introduced a transnational power that can implement its will within the territories of the member states without involving national authorities. The ratification of the Maastricht Treaty can thus be seen as an *extraordinary* political act that profoundly altered the constitutional order of both the EU and its member states. The ordinary representatives that signed (heads of states and governments) and ratified the Treaty (in most cases, parliaments) thereby acted as extraordinary representatives.

However, even if the concept of extraordinary representation is accepted, the EU cannot be characterised as the product of the will of a single constituent power. Jürgen Habermas (2012) has sought to overcome this problem through conceiving of the treaties as products of a *pouvoir constituant mixte* that consists of the citizens of Europe in a dual capacity: as citizens of the EU *and* of their respective member states. Notwithstanding the problem that citizenship is of course a constituted legal status, this highlights that if the concept of constituent power is to make sense in the EU context, it must be in the plural. The ECB (2002, p. 46; emphasis added) strikes a similar note in describing its foundations of authority: ‘It was the sovereign decision of the *peoples* of Europe (through their elected representatives) to transfer the competency for monetary policy and the other tasks enumerated in the Treaty to a newly created European body, and to endow it with independence from political interference’. The ECB thereby strikes a chord similar to that of legal scholars such as Dieted Grimm (2015, p. 48) and Miguel Poiares Maduro (2008, para. 21), who argue that the EU treaties are attributed to the peoples of Europe, not the governments or parliaments of the member states. In this account, the ECB, and the EU in general, derives its right to govern from the same source as the member states themselves. The will of the European peoples may have been conveyed by elected representatives, but it is the will that matters, not how it is represented.

One can dispute whether the ECB was ‘really’ the will of the peoples. Few peoples were asked, and the German Chancellor at the time, Helmut Kohl, later admitted that in forcing through Germany’s adoption of the euro he acted as a dictator (Paul, 2010, p. 293). While this may disqualify the ECB’s claim to be a product of the will of the peoples, it does not necessarily mean that the ECB’s public law does not operate on the assumption that it is. In a sense, the ECB’s extraordinary powers and its independence in exercising them *have* to be attributed to the constituent power of the peoples. That there are multiple constituent powers involved, however, raises certain problems.

The ECB takes the idea of central bank independence to its extreme conclusion. Through the effective constitutionalisation of the ECB’s price stability mandate (art. 127 TFEU) and independence from political actors at both the European and Member State levels (art. 130 TFEU), the ECB derives its right to govern monetary affairs from primary law. No constituted power can alter its status or mandate through ordinary legislation. This is intended to insulate it from political pressures that might compromise its single-minded pursuit of price stability. In principle, the ECB and its powers can be altered or withdrawn only by a new ‘sovereign decision by the peoples of Europe’. This means that unless the constituent power of any of the member state peoples is abrogated, leaving them no longer a people in the legal-political sense, all hold veto power over any change. The mandate of the ECB is thereby potentially even more rigid, and thus inherently conservative, than that of institutions subject only to a single constituent power.

The ECB’s independence means that no constituted powers can hold the ECB accountable for its acts and omissions. The ECB must ‘report’ to other constituted powers at the European level (art. 284(3) TFEU), but these institutions have no means of punishing it if they think it is failing its obligations. The ECB’s ‘input legitimacy’ is thus limited to the founding moment, and it is, through its ‘output legitimacy’ (informally) accountable only to the peoples who gave it its mandate.

The ECB’s constitutional status also reflects Sieyès’ (2014, p. 89) principle that ‘[n]o type of delegated power can in any way alter the conditions of its delegation’. Just as no other constituted power can alter the ECB, so it cannot alter its own mandate. It is thereby controlled by law and judicial review alone. It is thereby part of a system of checks and balances that is supposed to ensure that its governmental discretion is constrained by ‘a clear and limited mandate’ (Issing, 2002, p. 28) that it cannot control itself. This ‘clearly defined mandate’, according to the ECB (2002, p. 50), ‘lies at the very heart of the … “contract” between the people and the independent central bank’. It is an institution whose mandate, and the basic principles and values according to which it governs, are placed outside the ordinary political process by the founding act.

The practical and democratic consequences of the ECB’s constitutional position are wide-ranging. In ordinary times, it entails that the mandate of the central bank is almost impossible to adjust in accordance with changing macroeconomic values. Attributing the central bank’s mandate to the constituent power thereby attaches an inherently conservative bias to monetary policy, which at the same time constrains what member state authorities are able to do in terms of macroeconomic policymaking. The democratic legitimacy of this arrangement is questionable. It demands, at least, a strong popular attachment to the objective that the central bank pursues (in the case of the ECB: price stability).

The emergency situation raises further problems. The rigidity associated with the mandate in ordinary times is, in principle, carried over into the emergency situation. The central bank’s policymaking flexibility to address the crisis is thereby limited. The problem arises precisely because no constituted authority is empowered to alter, adjust or suspend the mandate. It is fixed between constituent moments. In a crisis, however, the restrictions of the mandate may threaten to exacerbate the crisis and prevent an effective response to it. As such, the central bank faces the age-old dilemma of emergency politics: honour the law but risk undermining the existence of the constituted order, or act beyond the mandate but violate the constitution. This was precisely what happened during the Eurozone crisis, which was understood as an existential crisis for the euro. At the peak of the crisis, the ECB famously stepped in to do ‘whatever it takes’ to rescue the euro. However, the acts that put this promise into practice – the so-called outright monetary transactions programme and the public sector purchases programme – violated one of the most fundamental principles of the ECB’s mandate (the art. 127 TFEU ban on monetary financing) and radically transformed and extended the powers of the ECB and its involvement in governing the Eurozone.

Due to its independence, no political authorities were involved in deciding on the ECB’s emergency political acts. There may, of course, have been support from governments across the Eurozone, but this was informal and ‘behind the scenes’. The ECB carries sole responsibility for acts that in effect transformed the constitutional construction of the Eurozone by, among other things, turning the ECB into a lender of last resort for the member states (see De Grauwe, 2013; Baldwin et al., 2015). This may be seen as a welcome development, but in introducing it the ECB itself effectively acted as an extraordinary representative of the constituent power. The absence of effective revolt against its acts may then be interpreted as a form of ‘acclamation by silence’ by the European people, in the name of whom the ECB now claims to act (see Lokdam, 2020).

This points to the problem with constructing the central bank as an institution of the constituent power. The constitutionalisation of the central bank’s mandate, in principle, restricts the flexibility of a central bank in dealing with unforeseen circumstances, because there are no institutionalised means of authorising (or punishing) new approaches to, or objectives for, monetary policy. The central bank as an institution of the constituent power is thereby (supposed to be) an inherently conservative power between moments of extraordinary politics. When the mandate proves untenable, however, the absence of institutionalised means of altering or suspending it means that the question of how to alter the mandate becomes opaque and inaccessible to democratic politics and contestation, as it did in the Eurozone crisis. If anything, then, the case of the ECB as an institution of the constituent power highlights the danger that rigid institutions of the constituent power present to a meaningful democratic politics.

Hjalte Lokdam

## Constituent power and the legislature

There is a strong case to be made that the whole purpose of the idea of constituent power was to limit the power of legislative assemblies. By the time Sieyès gave his famous account of constituent power in *What is the Third Estate?* there was already widespread agreement about what one might do to limit the power of the executive. First, ensure that the legislature possessed the power of the purse, making the executive financially dependent on the legislature. Second, give the legislature the power of impeachment, so that it could remove executive officials who violated the law. Finally, if these two first steps were insufficient, strictly separate executive and legislative officials so that the executive could not intervene in the legislative process at all.

But the question of how to limit the power of the legislature was more difficult. An executive capable of regularly checking the legislature was a frightening prospect and one that would inevitably violate the separation of powers. This would also introduce the prospect of endless stalemate and gridlock, a danger that might equally arise from having a second legislative chamber. As for the people themselves directly controlling legislative representatives through binding mandates, this would make it impossible for the legislature to serve as a space for national deliberation.

Sieyès’ idea of constituent power was a way out of this bind. It was a strategy for taming the legislature that did not require mandates, a strong executive, a senate or a house of lords. Instead of being directly checked and controlled by any of these other agents, the powers of the legislature would be made strictly subject to a constitution that it was unable to change or amend. The constitution could only be changed or amended through an entirely different process that was separate from the normal process of passing legislation, and which ideally would involve an entirely different representative body. Sieyès declared to the French National Assembly:

We have as a fundamental and constitutional principle that the ordinary legislature will not be able to exercise the constituent power…the ordinary National Assembly will not be more than a legislative assembly. It will be forbidden from every touching any part of the Constitution. When it will be necessary to review and reform some part, it is to be done by an express Convention, limited to this unique object, that the Nation will decree the changes that appear to it useful to make to the Constitution (Sieyès, 1789. p. 19).

Limited in this way by a constitution, and unable to exercise the constituent power, the legislative assembly would be incapable of infringing on the rights of citizens.

These reflections on the origins of the concept of constituent power might lead one to suppose that this concept would be of little value for thinking about legislatures in contemporary politics. After all, most legislative assemblies today are limited – whether through a written constitution that it is beyond the power of the legislature to change, as Sieyès proposed, or through the sort of checks that he opposed. Yet I want to suggest that this is not the whole story. If the constituent power were to become widely accepted as the grounds of political authority, the power and prominence of legislatures would be likely to radically increase.

If constituent power was originally a way to tame the power of the legislature, it also raised the practice of the legislature, the practice of representatives coming to a decision through deliberation and parliamentary procedure, to new heights of importance. As Jack Rakove noted about the constitutional convention that wrote the United States Constitution in 1787, ‘the politics of the Convention resemble that of any legislative body, and its votes become grist for the fine-milling techniques of roll-call analysis that are commonly used to explain decision-making in Congress’ (1996, p. 15). The state assemblies that ratified the Constitution resembled legislative bodies even more than the original Convention did – and this is even more true of the French National Assembly which, to Sieyès’ dismay, was forced to engage in actions that went well beyond the limited role of an ideal constituent assembly. This is a trend that has lasted throughout the modern era: the Weimar National Assembly of 1919 and the Indian Constituent Assembly were among the many instances of large representative bodies exercising a function for constituent power.

According to Sieyès, deliberation by a group of representatives was essential to carrying out the task of the constituent power and formulating a constitution. A constitution must be a national act, which is made in the general interest. Yet the general interest was not obvious or given in advance. It was necessarily a composite of countless smaller particular interests. The only way the general interest could be achieved was through a consensus enacted by deliberation:

In every deliberation there is a kind of problem to be solved. This is to know, in any given case, what the general interest would prescribe. When the discussion begins, it is not possible to identify the direction it will take to reach the discovery with certainty…hence the clash and coincidence of opinions….All these individual interests have to be allowed to jostle and press against one another, to take hold of the question from one point of view, then another, each trying to push it according to its strength towards some projected goal. In this trial, views that are useful and those that are harmful will be separated from one another. Some will fall while others will maintain their momentum and will balance one another until, modified and purified by their reciprocal interaction, they will end up becoming reconciled with one another…just as in the physical universe a single more powerful movement can be made up of a multitude of opposing forces (Sieyès, 2003, p. 39-40).

Sieyès’ use of the concept of constituent power and his opposition to the idea of popular sovereignty are related to this approach to achieving the general interest. Whereas the concept of popular sovereignty makes the will of the people, usually as evinced by a plebiscite, the underlying source of legitimacy for a constitution, the concept of constituent power makes the creation of a constitution one particular task or function, which is to be carried out by representatives like other tasks or functions. This is difficult to justify unless one thinks that creating a constitution is an act that requires bringing together the representatives of different interests and perspectives who can arrive at a decision in the general interest.

Although the convention that exercises the constituent power is not the normal legislative assembly, it is inevitably a body with some resemblance to a legislative assembly, and which deliberates in a somewhat similar fashion. And although the normal legislative assembly does not exercise the constituent power, it somehow approximates the sort of political deliberation that occurs in the act of constitutional founding. The various interests and opinions that go into a constituent assembly are unlikely to disappear – they are likely to also be represented in the normal legislature, meaning there will be some continuity between the debates in the constituent assembly and normal legislative debates.

According to the general idea of popular sovereignty, the people which rules delegates power to different political actors – to executive, legislative and judicial officials – none of which can claim to speak for the people definitively. In practice, however, it tends to be the executive that expresses the best claim to represent the people, since the choice of a president or prime minister comes closest to being a decision in which the whole nation is involved.

If constituent power were to become widely accepted as the grounds of constitutional legitimacy, this situation might change radically. It would remain the case that no ordinary power – executive, legislative or judicial – could claim to speak for the people definitively. Yet the legislature would arguably have a better claim to do so than the other powers. It alone among the constituted powers can plausibly attempt to achieve a ‘general interest’, since it is the only constituted power that allows a range of ‘individual interests…to jostle and press against one another, to take hold of the question from one point of view, then another, each trying to push it according to its strength towards some projected goal’ (Sieyès, 2003, pp. 39–40).

The concept of constituent power would alter the relationship between the legislature and the people no less radically. It is easy to presume, when thinking in terms of popular sovereignty, that the people are simply the masters of their representatives – after all, it is the people who possess sovereignty, their representatives are merely those delegated by them to carry out tasks that they wish. The concept of constituent power renders this relationship significantly more complex, since it denies that the people have the kind of final authority that goes with sovereignty. Put another way: the electorate that chooses the members of the legislature is itself a constituted power. It is not prior to constituted institutions, as we might think a sovereign people is, but is constituted like them through the constitution. The selection of representatives to the legislature is merely one moment in the process of law-making, accomplished by one constituted power among several. It is not a moment that reveals the sovereign will of the people. If Sieyès sought, through his concept of constituent power, to limit the power of the legislature, he also limited the claim to power of the electorate, and thus in another way preserved a degree of autonomy for the legislature that is harder to justify in a system based on popular sovereignty.

This contribution has been something of a thought experiment. If we were to rethink the foundations of political legitimacy in terms of constituent power rather than popular sovereignty, where might it lead with respect to the role of the legislature? And the somewhat unexpected conclusion, given Sieyès’ intention, is that this could very well lead to legislatures that are more prominent and more autonomous of the electorate. Yet I suspect that this is also why constituent power is unlikely to become our dominant political conception any time soon. In her important recent book *Constituent Power: A History* (2020), Lucia Rubinelli has documented how Sieyès’ distinction between constituent power and sovereignty was lost in the twentieth century. Since Carl Schmitt, constituent power has been increasingly interpreted as no different from sovereignty. But we might ask whether this is because the sort of politics that constituent power tended toward has become increasingly unrealistic. On the other hand, the fact that this concept is still part of our political language, and that constitutional conventions remain an important institution, suggests that we should not entirely discount constituent power and the kind of politics Sieyès envisioned either.

Will Selinger

## Notes

1. This one was the first case of a *saisine parlementaire* (the ex-ante referral by at least 60 members of the Parliament) introduced in France by the constitutional reform of 1974.
2. Notice that the Council deciding for the compatibility was siding with the majority of the Parliament, whereas the public opinion was divided: 48% were favorable to abortion.
3. Schmitt uses the category of *Bund* instead of *Föderation* for federal political orders, which extends beyond statehood and includes international federal orders of a lasting and constitutionalized kind.
4. As Nicholas Aroney has shown, constitutional amendment in federations often requires only a majority decision, not unanimity – which may raise questions on the legitimacy of such pre-regulated transformations. The Swiss constitution, in contrast, requires a popular referendum for an amendment to the constitution.
5. The federal order could only be abolished through revolution – or through the resolution on a new constitution, the procedural pathway towards which is not elaborated on in the relevant article 146 of the Basic Law.
6. For disintegrative action, such as in the case of Malaysia, one could also speak of ‘destituent power’, although the term is, at the moment, still claimed for a number of phenomena (cf. Patberg, 2019). Ultimately, though, re-constituent and destituent power could be conceptualized as sub-species of constituent power.
7. First substantiated by Anne Peters, the idea of a *pouvoir constituant mixte* has been applied by Habermas to the EU constellation of a dual role of all EU citizens in the legitimatory process (Habermas, 2011).
